# Exosomal miR-135a derived from human amnion mesenchymal stem cells promotes cutaneous wound healing in rats and fibroblast migration by directly inhibiting LATS2 expression

**DOI:** 10.1186/s13287-020-1570-9

**Published:** 2020-02-13

**Authors:** Shaoying Gao, Tao Chen, Yi Hao, Feifei Zhang, Xiujun Tang, Dali Wang, Zairong Wei, Jianping Qi

**Affiliations:** Department of Plastic Surgery, Affiliated Hospital of Zunyi Medical University, Zunyi, Guizhou People’s Republic of China

**Keywords:** Amnion mesenchymal stem cells, Exosomes, LATS2

## Abstract

**Background:**

Wound healing is a complex pathophysiological process that involves a variety of cells and cytokines. In this study, we found that local injection of human amnion mesenchymal stem cells into wounds in rats could promote wound healing. Therefore, we hypothesized that the exosomes of human amnion mesenchymal stem cells contain substances that regulate the migration of epidermal cells. It has been reported that miR-135a is involved in cell migration and transformation. However, there have been no reports of its function in skin wound healing.

**Methods:**

To test this hypothesis, we injected exosomes overexpressing miR-135a directly into the wound margin. In addition, we tested the migration of BJ cells with overexpression or knockdown of miR-135a in vitro. Additionally, Western blot analysis was used to detect the expression of fibroblast migration-associated proteins after treatment with miR-135a overexpression or knockdown.

**Results:**

MiR-135a significantly promoted wound healing compared to the control treatment. Western blot analysis showed a significant downregulation of LATS2 after overexpression of miR-135a. In addition, knockdown of miR-135a effectively attenuated the promoting effect of exosomes on cell migration.

**Conclusions:**

Our results indicated that miR-135a promotes wound healing, which may be mediated by downregulating LATS2 levels to increase cell migration. This study provides a rationale for the therapeutic effect on wound healing of miR-135a in exosomes derived from human amnion mesenchymal stem cells.

## Introduction

Skin wound healing is a complex process involving a number of highly coordinated steps, mainly divided into inflammatory response, epithelialization and wound contraction, collagen deposition, and remodeling [1–3]. How to promote wound healing is an urgent problem to be solved in clinical practice, and it is also the focus of long-term research by scholars worldwide. Although some progress has been made in the study of wound healing, there are still various problems in clinical application, and the curative effect is suboptimal. Therefore, it is very important and meaningful to find targets for promoting wound healing. The main mechanism of the skin formation process is the migration and proliferation of keratinocytes. During migration, keratinocytes undergo a process similar to epithelial-mesenchymal transition (EMT) during migration, which is characterized by a weakened connection between keratinocytes and between cells and the basal layer [4]. The cell migration ability is enhanced, and the wound surface is contracted [5]. Numerous experimental results have confirmed that promoting keratinocyte migration can accelerate wound healing.

MicroRNAs are a class of short noncoding RNAs that function by posttranscriptional regulation of protein expression [6]. Some microRNAs play an important role in wound healing and fibrotic diseases [7, 8]. The literature indicates that miR-203 and miR-210 promote wound healing by promoting keratinocyte migration and proliferation [9, 10], respectively, while the miR-99 and miR-200 families delay wound healing by inhibiting keratinocyte migration [11]. Current research indicates that miR-135a can promote the migration abilities of breast cancer cells [12]. However, there have been no reports of its function in skin wound healing. Therefore, it is necessary to carry out systematic and in-depth research on these problems, which is of great significance for clarifying the mechanism of wound healing and exploring new targets for clinically promoting wound healing.

In this study, we revealed an important role for miR-135a in promoting BJ cell migration and wound healing in vitro. Our results indicated that miR-135a could promote wound healing by accelerating cell migration and had a better wound healing effect in animal models. In addition, our data indicated that miR-135a-mediated downregulation of LATS2 (large tumor suppressor 2) increased the migration of BJ cells. In summary, our study provides a basic elucidation of the molecular mechanisms of wound healing and a theoretical basis for the therapeutic effect of exosomal miR-135a on wound healing.

## Materials and methods

### Animals and ethics statement

Forty adult SD rats weighing 200 ± 50 g were obtained from the Model Animal Research Center of Chongqing Medical University. Rats were randomly divided into two sets of animal experiments, 20 in each set. Rats were anesthetized by intraperitoneal injection of 3% sodium pentobarbital (35 mg/kg). The back skin of rats was shaved and disinfected and cut 1 cm apart on both sides of the midline in the rat’s back. A full-thickness skin defect wound (1.5 cm × 1.5 cm) was prepared on the left and right sides, and the area was approximately 2.25 cm^2^. Animal experiments were fully compliant with the Guidelines for the Care and Use of Laboratory Animals, and the protocol used in this study was approved by the Animal Care Committee of Zunyi Medical University.

### Animal wound healing experiments

Twenty rats were used for each set of experiments. The first set of animals was randomly divided into four groups: (1) saline control group (*n* = 5), (2) hAMSC (human amnion mesenchymal stem cell) low-density group (*n* = 5), (3) hAMSC medium-density group (*n* = 5), and (4) hAMSC high-density group (*n* = 5). Type I collagen (0.5 ml) was prepared and mixed with hAMSCs at 1 × 10^4^ cells/ml, 1 × 10^5^ cells/ml, and 1 × 10^6^ cells/ml, and multipoint injection was applied to each wound (injection point was 1 mm from the midpoint of each side of the wound edge). In the control group, an equal amount of physiological saline was injected. The second set of animals was randomly divided into five groups: (1) saline control group (*n* = 5), (2) 293 T-Exo group (*n* = 5), (3) hAMSC-Exo (human amnion mesenchymal stem cell exosome) group (*n* = 5), (4) hAMSC-miR-135a OE group (*n* = 5), and (5) hAMSC-miR-135a KD group (*n* = 5). The mode of administration was such that the type I collagen was mixed with the exosomes and uniformly coated on the fresh sterile wound surface on the left front side of the rat’s back. All wounds were covered with Vaseline gauze. The wound size was measured and analyzed over time using Image-Pro Plus 6.0 software. Skin tissue samples were collected at day 15 for further histological analysis. Healing index = (1 − wound area/original area) × 100%.

### Histological examination

Skin samples were collected on the 15th day, fixed, dehydrated, embedded in paraffin, and then cut into 4 μm thick sections, followed by hematoxylin-eosin staining (HE) as previously described. Images of stained sections were obtained by an FSX100 microscope.

### Cell culture and isolation of hAMSC-Exos

hAMSCs and BJ cells were obtained from Shanghai Institute of Biochemistry and Cell Biology. Both were cultured in DMEM (Gibco) supplemented with 10% exosome-depleted FBS and 1% antibiotic-antimycotic solution in a humidified incubator at 37 °C with 5% CO_2_.

For isolation of hAMSC-Exos, 1 × 10^6^ hAMSCs were inoculated into T75 flasks in DMEM supplemented with 10% exosome-depleted FBS for 48 h; hAMSC medium was collected and centrifuged at 300*g* for 10 min. After centrifugation, the supernatant was collected and filtered through a 0.22-μm filter to remove cell debris. The remaining supernatant was then ultracentrifuged with a Ti70 rotor at 120,000*g* for 10 h. The exosome-enriched pellet was obtained and resuspended in a small amount of PBS, and then the protein content was measured by a BCA protein assay kit. The concentration was adjusted to 40 μg/mL and stored at − 80 °C. hAMSC-Exos were examined to confirm their characteristics using a nanoparticle tracking analyzer, transmission electron microscopy, and Western blotting.

### Fibroblast migration analysis

Fibroblasts were subjected to a conventional scratch test. Briefly, fibroblasts were seeded at a density of 1 × 10^6^ cells in 35-mm culture dishes and starved for 12 h in serum-free DMEM. The tip of a pipette was used to create a cross-shaped scratch in the middle of each well. The cells were then gently washed with PBS followed by different treatments by incubation in an air atmosphere of 37 °C and 5% CO_2_ for 24 h. Images were acquired over time. The gap area was measured and recorded and then compared to the initial gap size at 0 h by Image-Pro Plus 6.0 software. The migration area was calculated as (%) = ((original gap area − gap area at *X* h)/original gap area) × 100%. Migration of fibroblasts was also determined by the Transwell assay using an 8-μm pore filter. Approximately 1 × 10^6^ fibroblasts were seeded into the upper compartment, while exosomes with different treatments were added to the lower compartment. Cells were cocultured for 24 h; nonmigrating cells in the upper chamber were wiped off, and the remaining cells were stained with 0.4% crystal violet.

### Western blot

The protein concentration was measured by the BCA method and quantified. The experimental procedure was carried out according to a conventional Western blotting protocol. The proteins were separated using 10% SDS-PAGE and transferred to a PVDF membrane under constant current of 320 mA. The membrane was blocked with 5% skim milk at room temperature for 1.5 h and with 1:1000 dilutions of anti-LATS2 (Abcam), anti-E-cadherin (Abcam), anti-N-cadherin (Abcam), anti-α-SMA (Alpha-smooth muscle actin) (Abcam), anti-CD9 (Abcam), anti-CD63 (Abcam), and anti-CD81 (Abcam) overnight at 4 °C. The next day, the membrane was washed three times with TBST and incubated with a 1:1000 diluted HRP-conjugated secondary antibody (Abcam) for 1 h at 37 °C. After washing three times with TBST, chemiluminescence was performed using an ECL reagent (Bio-Rad). The band intensity for each protein on the membrane was scanned by a scanner and analyzed by image processing software.

### Real-time PCR

The expression levels of individual miRNAs were ascertained using RT-PCR. The PrimeScript™ RT Reagent Kit (Jiangsu Synthgene Biotechnology Co., Ltd) was used to synthesize cDNA. SYBR Green qPCR assay (Jiangsu Synthgene Biotechnology Co., Ltd) was used to detect the expression of miR-135a and LATS2. PCR was performed for 45 cycles (95 °C, 10 s; 60 °C, 30 s) after an initial denaturation step (95 °C, 5 min) on the Bio-Rad CFX96 system. The expression levels of miRNAs and mRNAs were quantified using the 2^−ΔΔCT^ method, and miR-16-5p and GAPDH were used as the internal controls for miRNA and mRNA, respectively. All reactions were performed in triplicate. Primers were synthesized by Applied Biosystems (Table 1).

**Table 1:** Primers for real-time PCR

Primers	Sequences
miR-135a	F 5′-ACACTCCAGCTGGGTATGGCTTTTTATTTCCT-3′
	R 5′-GGTGTCGTGGAGTCGGCAA-3′
LATS2	F 5′-AGCCATGTCAGACAGGACAGCAT-3′
	R 5 ′-CTCGAAGAGAATCACTCCAACACTC-3′
miR-16-5p	F 5′-TAGCAGCACGTAAATATTGGCG-3′
	R 5′-TGCGTGTCGTGGAGTC-3′
GAPDH	F 5′-GCACCGTCAAGGCTGAGAAC-3′
	R 5′-ATGGTGGTGAAGACGCCAGT-3′

### Luciferase assay

Cells (1 × 10^6^) were seeded in triplicate in 6-well plates and allowed to settle for 24 h. The luciferase plasmid pGL3-LATS2-3′UTR (wild-type/mutant, wt/mut) or control luciferase plasmid plus 1 ng pRL-TK Renilla plasmid were transfected into hAMSCs using Lipofectamine 2000 (Thermo) according to the manufacturer’s recommendation. Luciferase and Renilla signals were measured 48 h after transfection using a Dual Luciferase Reporter Assay Kit according to the manufacturer’s protocol.

### Statistical analysis

All statistical analyses were performed by GraphPad Prism 7.0 software. Student’s *t* test was used to analyze significant differences in this study. The error bars indicate the standard deviation from the mean of triplicate measurements. Asterisks indicate significant differences (*p < 0.05; **p < 0.01; ***p < 0.001) compared with the corresponding control.

## Results

### HAMSC can promote wound healing and wound epidermalization of full-thickness skin defects in the backs of rats

**Fig. 1:**
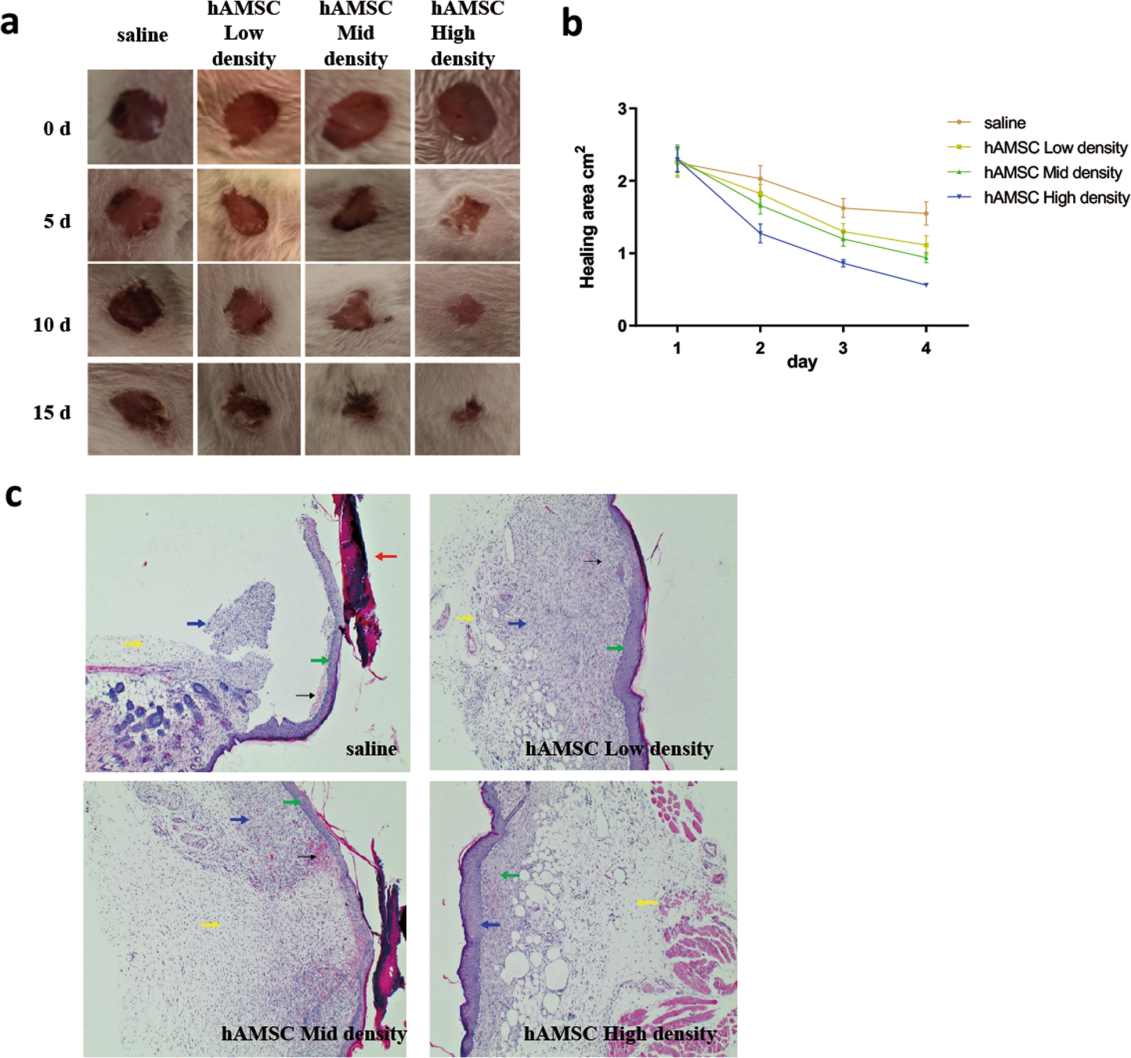
hAMSCs promoted skin wound healing in rats. **a** Full-thickness excision wounds were made on the backs of SD rats on day 0 (D0). The wound healing progress was digitally photographed on D0, D5, D10, and D15 post-wounding in both the control and hAMSC treatment groups. Ruler scale = 1 mm. **b** The healing area was calculated at the given time point relative to the original healing area on D0. *n* = 5. **c** The effect of hAMSCs on the morphological change of skin wounds was assessed by HE staining. Dotted lines indicate the newly formed epithelial tongue. Scale bar = 100 μm

The wound healing experiments showed that the wound healing rate of the hAMSC injection group increased dependent on the cell density compared with that of the control group (Fig. 1a). The wound area of the hAMSC high-density group was significantly lower than that of the control group on the 5th day (Fig. 1b). The results showed that hAMSCs could promote wound healing. Wound epidermalization plays a decisive role in wound healing, is related to the speed of wound healing, and is the standard for testing wound healing. After the 15th day, the HE staining results of wound tissues were observed (Fig. 1c). In the saline group, there was damaged skin with jagged edges, more subcutaneous hemorrhage (black arrows), scab formation on the damaged skin (red arrows), thinner dermis hyperplasia (green arrows), a small amount of inflammatory cells (yellow arrows), and a small amount of new granulation tissue (blue arrows). With the increase in density of hAMSCs, the damaged surface of the skin gradually healed, subcutaneous hemorrhage gradually decreased, dermis gradually proliferated, inflammatory cells gradually decreased, and new granulation tissue gradually increased. These results indicate that hAMSCs could promote wound epithelialization.

### Isolation and characterization of hAMSC-Exos

**Fig. 2:**
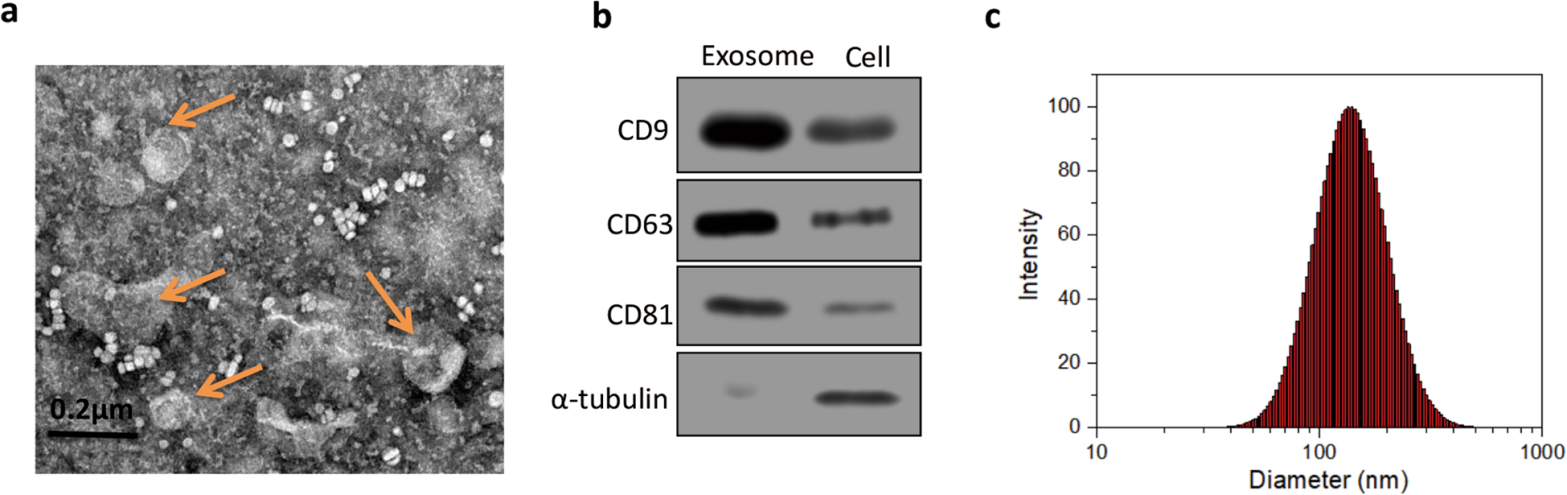
Characterization of hAMSC-Exos. **a** TEM image of hAMSC-Exos (*n* = 5). **b** Western blot analysis of exosomal markers CD9, CD63, and CD81 (*n* = 3). **c** Size distribution of hAMSC-Exos (mean diameter = 103 nm, *n* = 3)

By TEM observation, the exosomes were circular or elliptical in shape and had a diameter of approximately 30 to 150 nm (Fig. 2a). Western blot results further confirmed that the exosomal markers CD9, CD63, and CD81 were expressed in hAMSC-Exos (Fig. 2b). The particle size distribution was measured by a nanoparticle tracking analyzer (Fig. 2c), and the results showed that 90% of the particles were distributed between 30 and 150 nm (average diameter = 103 nm).

### Detection of miR-135a expression in exosomes

**Fig. 3:**
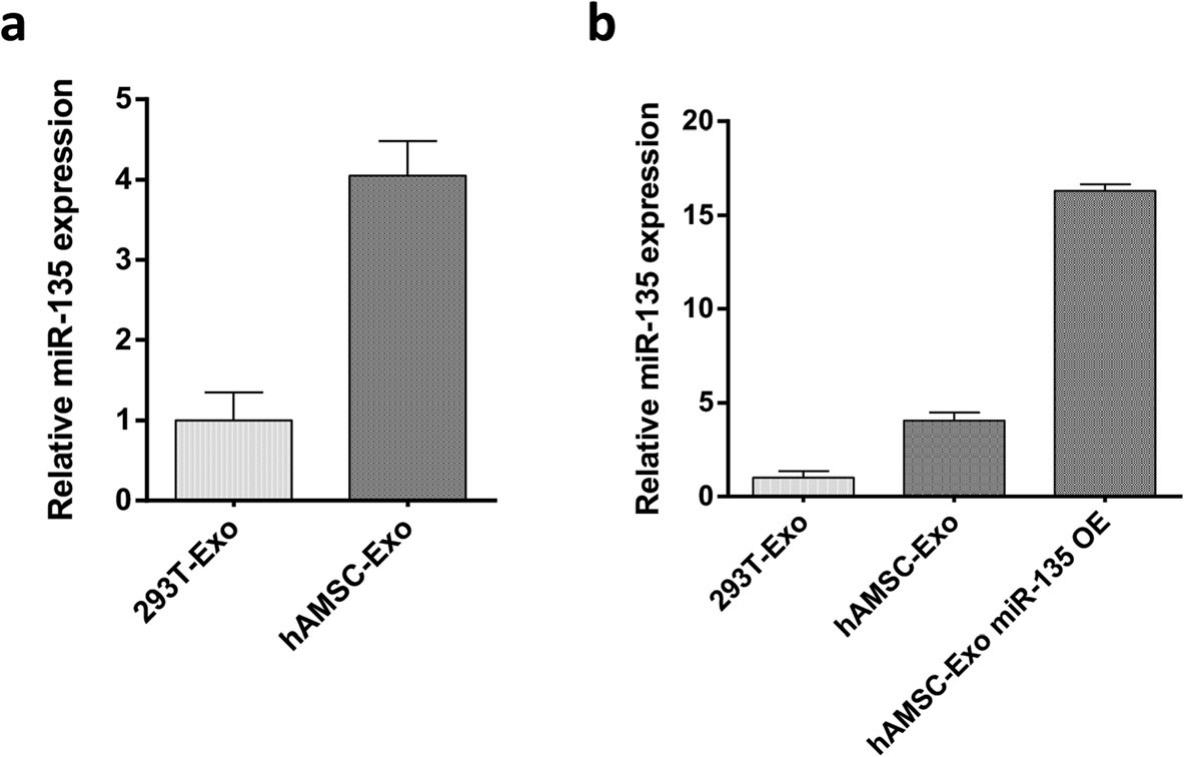
Relative miR-135 expression in exosomes. **a** Relative miR-135 expression in 293 T- Exo and hAMSC-derived exosomes (*n* = 5). **b** Relative miR-135 expression in exosomes from 293 T cells, hAMSCs, and hAMSCs overexpressing miR-135a (*n* = 5)

The expression of miR-135a in exosomes was detected by real-time PCR. By detecting the microRNA expression levels in exosomes in both cell lines (293 T and hAMSC), we found that the expression level of miR-135a was higher in hAMSC (Fig. 3a). In addition, we overexpressed miR-135a in hAMSCs, which resulted in a corresponding increase in miR-135a in exosomes (Fig. 3b).

### HAMSC-Exos promote migration in BJ cells

**Fig. 4:**
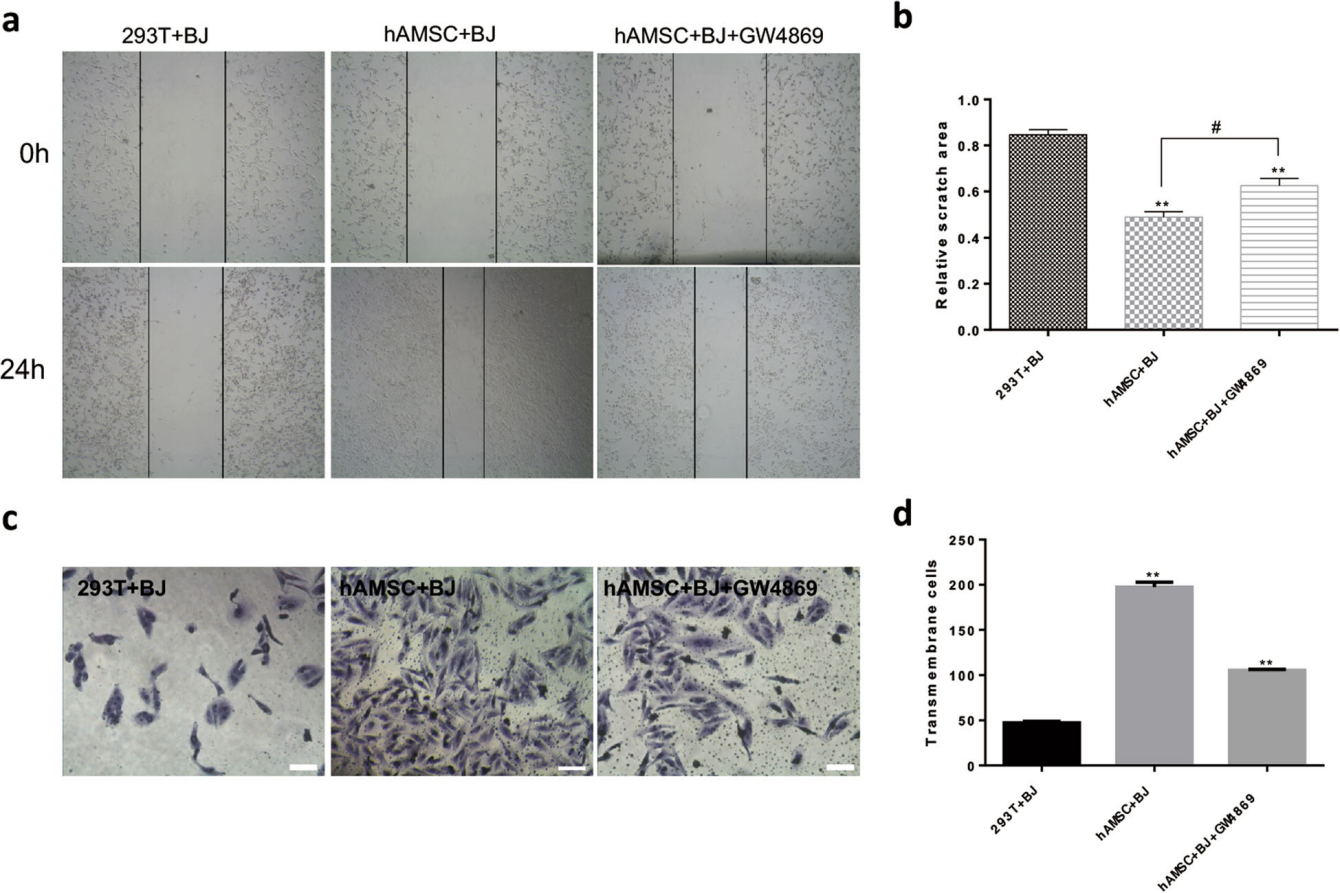
Effects of hAMSC-Exos on the migration of BJ cells. **a** Representative images of the scratch wound healing assay of cells treated with hAMSC-Exos at different time points. **b** Quantitative analysis of cell migration. **p < 0.01 compared to the control group, ^#^*p* < 0.05 (*n* = 5). **c** Microscopic view of migrated cells after crystal violet staining. Scale bar = 50 μm. **d** Quantitative analysis of the migrated cells. **p < 0.01 compared to the control group (*n* = 5)

The cell scratch assay (Fig. 4a) and the Transwell cell migration assay (Fig. 4c) were used to determine the effect of hAMSC-Exos on cell migration. As shown in Fig. 4b, the hAMSC group and the GW4869-treated hAMSC group significantly enhanced the migration of fibroblasts to the scratch area after 24 h compared to the control group. There were also significant differences between the hAMSC group and the GW4869-treated hAMSC group. Similar results were obtained by the Transwell assay (Fig. 4d), and the hAMSC group and the GW4869-treated hAMSC group had significantly enhanced cell migration compared to the control group. Taken together, these results indicated that exosomes from hAMSCs play a key role in accelerating BJ cell migration.

### HAMSC-Exos regulate the expression of migration-associated proteins

**Fig. 5:**
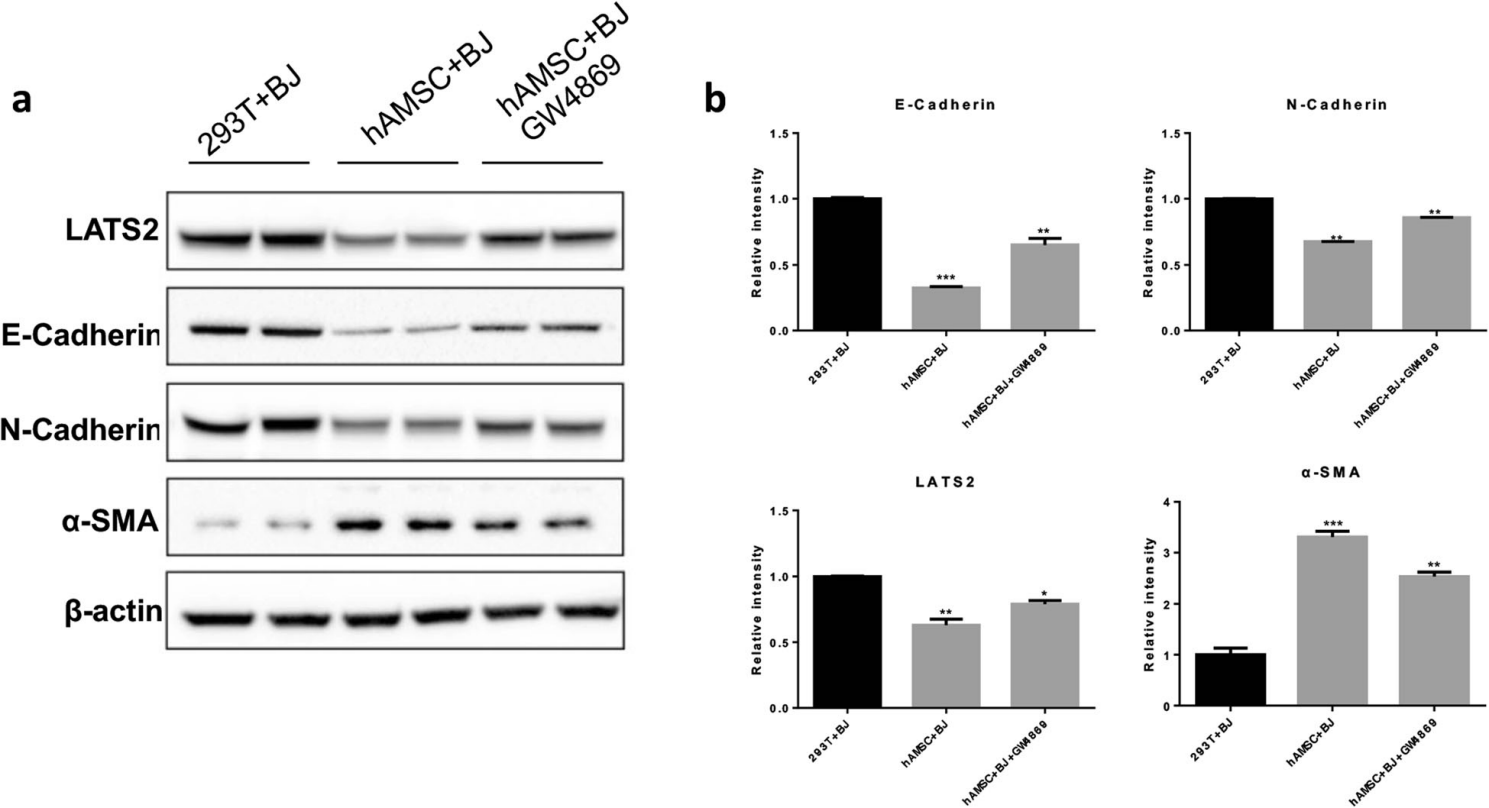
Effects of hAMSC-Exos on the expression of corresponding proteins involved in cell migration. **a** Protein expression levels of E-cadherin, N-cadherin, LATS2, and α-SMA after treatment with hAMSC-Exos were detected using Western blot. **b** Histogram summarizing the results in **a**, **p<0.01, ***p<0.001 compared to the control group (*n* = 3)

Western blot analysis (Fig. 5a) was used to determine the effect of hAMSC-Exos on cell migration-associated proteins. As shown in Fig. 5b, the hAMSC group and the GW4869-treated hAMSC group significantly downregulated the expression of the cell migration-associated proteins E-cadherin, N-cadherin, and LATS2 after 24 h of treatment compared with the control group. After treatment with GW4869 (an exosome inhibitor), the downregulation of these proteins elicited by hAMSC-Exos was attenuated. The expression of α-SMA protein was significantly increased in the hAMSC group and the GW4869-treated hAMSC group, indicating that hAMSC-Exos promoted cell migration. Taken together, these results indicated that exosomes from hAMSCs could promote BJ cell migration by regulating the expression of migration-associated proteins.

### MiR-135a can promote wound healing and wound epidermalization of full-thickness skin defects in the backs of rats

**Fig. 6:**
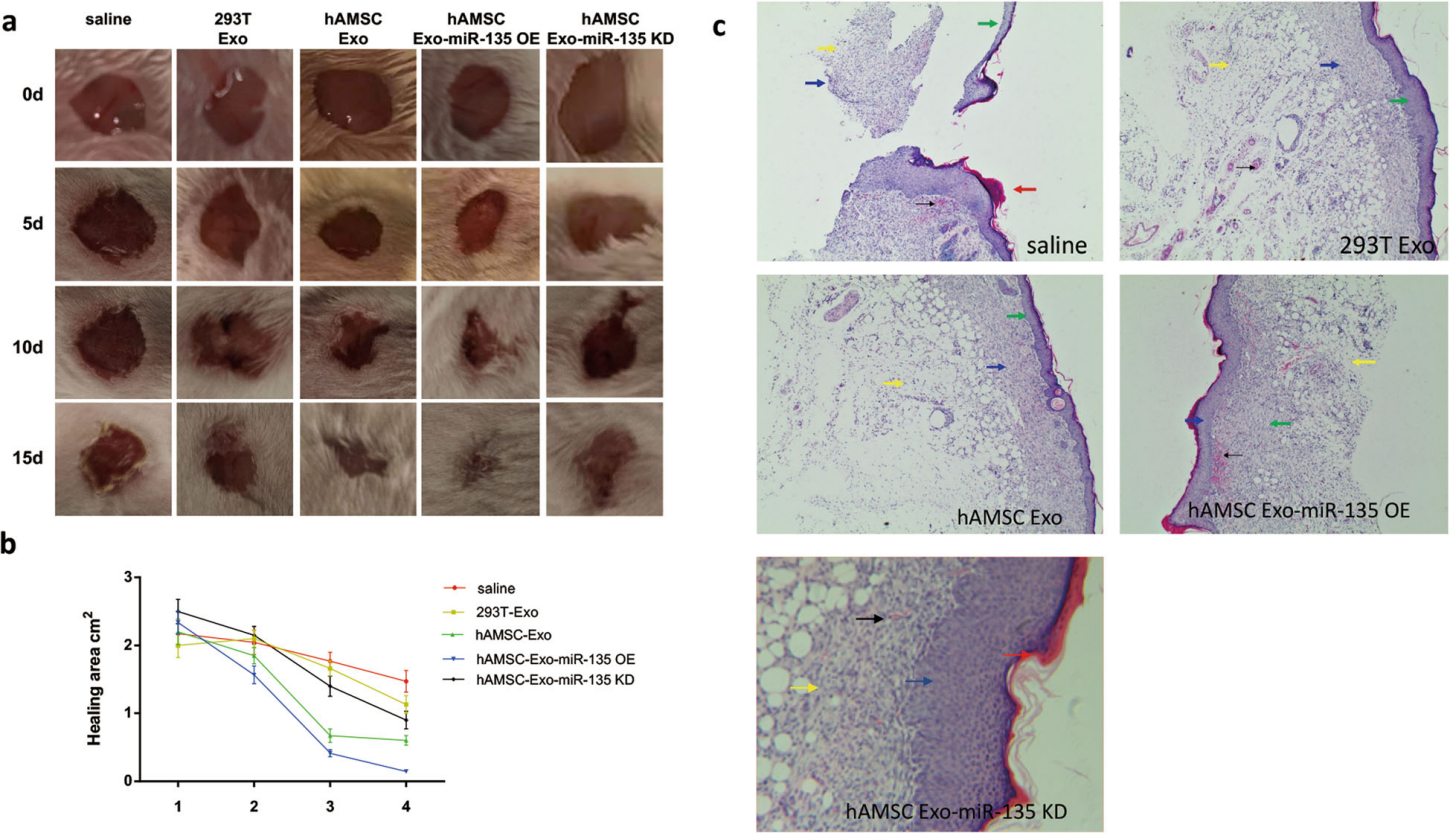
MiR-135a promoted skin wound healing in rats. **a** Full-thickness excision wounds were made on the backs of SD rats on day 0 (D0). The wound healing progress was digitally photographed on D0, D5, D10, and D15 post-wounding in both the control and hAMSC-Exo treatment groups. Ruler scale = 1 mm. **b** The healing area was calculated at the given time point relative to the original healing area on D0. *n* = 5. **c** The effect of miR-135a on the morphological change of skin wounds was assessed by HE staining. Dotted lines indicate the newly formed epithelial tongue. Scale bar = 100 μm

The wound healing experiments showed that the miR-135a overexpression group had the fastest wound healing rate compared with the other groups (Fig. 6a), and the wound area was significantly smaller on the 5th day than that in the control group (Fig. 6b). However, the wound healing rate of the miR-135a knockdown group was significantly lower than that of the hAMSC-Exo group. Similarly, HE staining was observed after the 15th day (Fig. 6c). Compared with that of the control group, the skin damage surface of the miR-135a group healed better, the skin dermis layer proliferated, and a large amount of new granulation tissue was observed. The number of inflammatory cells decreased in the miR-135a overexpression group. The results showed that miR-135a in exosomes could promote wound healing and wound epithelialization.

### MiR-135a promotes BJ cell migration by regulating LATS2

**Fig. 7:**
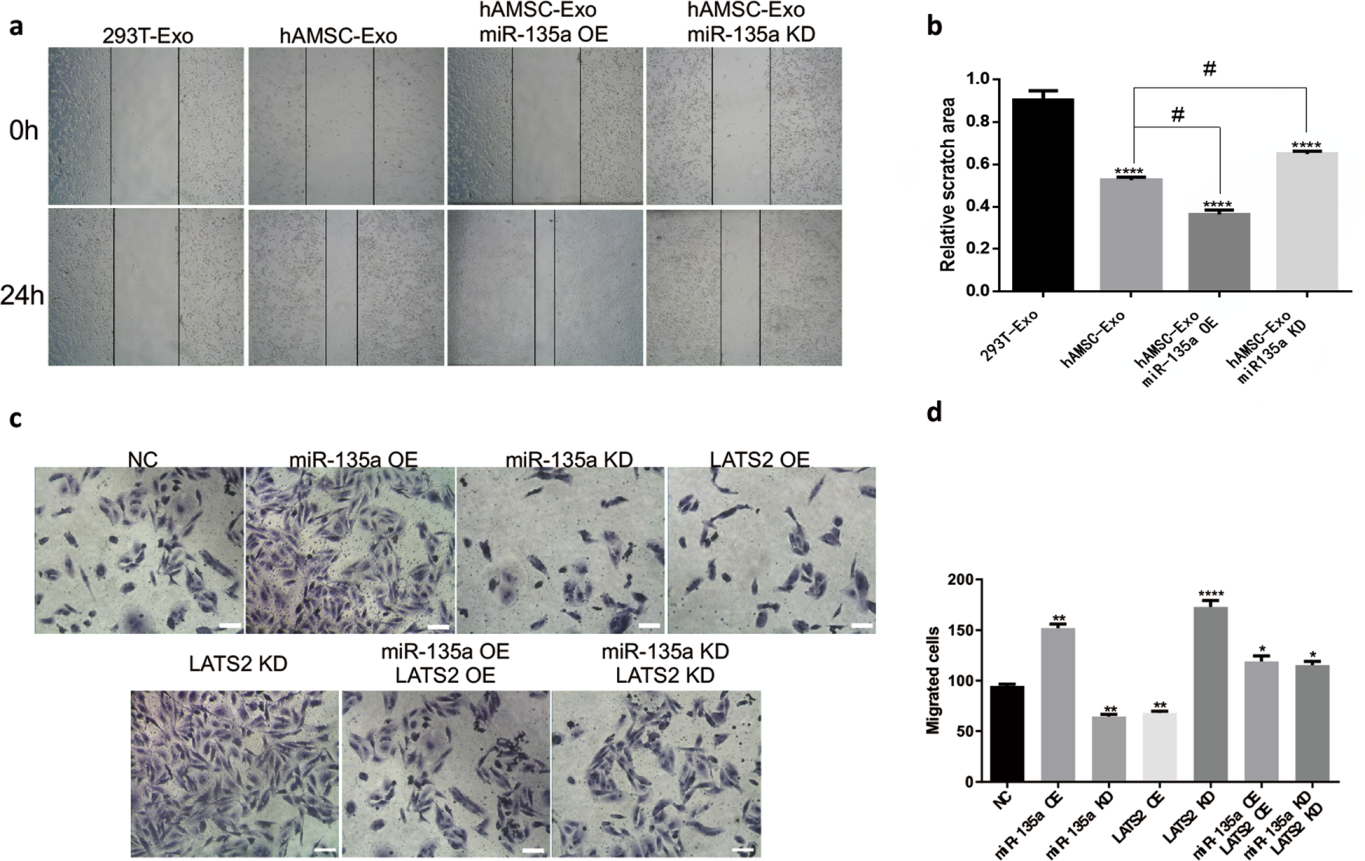
Effects of miR-135a on the migration of BJ. **a** Representative images of the scratch wound healing assay treated with hAMSC-Exos at different time points. **b** Quantitative analysis of fibroblast migration. ****p < 0.0001 compared to the control group, ^#^*p* < 0.05 (*n* = 5). **c** Microscopic view of migrated cells after crystal violet staining. Scale bar = 50 μm. **d** Quantitative analysis of transmembrane cells. **p < 0.01 compared to the control group (*n* = 5)

The cell scratch assay (Fig. 7a and b) was used to determine the effect of miR-135a on BJ cell migration. As shown in Fig. 7b, each group treated with hAMSC-Exos had significantly enhanced cell migration to the scratch area after 24 h of treatment compared to the control group. Moreover, the miR-135a overexpression group had the strongest ability to promote cell migration, and the miR-135a knockdown group impaired the cell-promoting ability of hAMSC-Exos. Transwell assays (Fig. 7c, d) confirmed that miR-135a could promote BJ cell migration by regulating LATS2. Figure 7d shows that overexpression of miR-135 or knockdown of LATS2 can promote cell migration, while knockdown of miR-135 or overexpression of LATS2 can inhibit cell migration. The recovery experiment further indicated that miR-135a in hAMSC-Exos plays a key role in accelerating BJ cell migration by regulating LATS2.

### MiR-135a can regulate the expression of BJ cell migration-related proteins

**Fig. 8:**
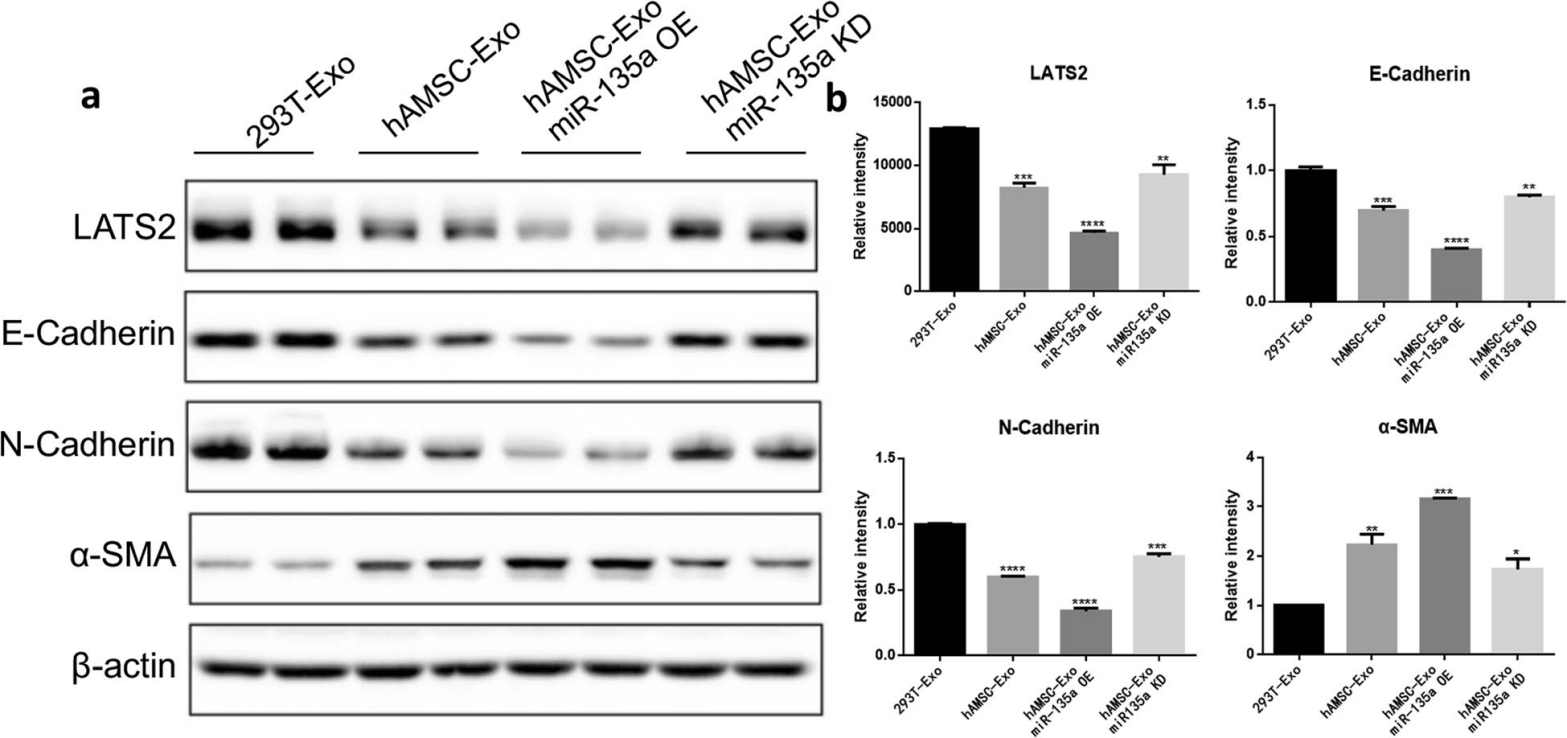
Effects of miR-135a on the expression of corresponding proteins involved in BJ cell migration. **a** Protein expression levels of E-cadherin, N-cadherin, LATS2, and α-SMA after treatment with hAMSC-Exos were detected using Western blot. **b** Histogram summarizing the results in **a**, **p < 0.01, ***p < 0.001, ****p < 0.0001 compared to the control group (*n* = 3)

Western blot analysis (Fig. 8a) was used to determine the effect of miR-135a on BJ cell migration-associated proteins. As shown in Fig. 8b, after treatment for 24 h, the groups treated with hAMSC-Exos significantly downregulated the expression of the cell migration-associated proteins E-cadherin, N-cadherin, and LATS2. The downregulation effect of the miR-135a group was most pronounced. After knocking out miR-135a, hAMSC-Exos had a reduced ability to downregulate these proteins. The expression of the α-SMA protein was significantly increased in each group treated with hAMSC-Exos, indicating that hAMSC-Exos promoted the migration of fibroblasts and that miR-135a played a key role in this process. Taken together, these results indicated that miR-135a in hAMSC-Exos regulates the expression of migration-associated proteins and promotes cell migration.

### MiR-135a directly targets LATS2 in fibroblasts

**Fig. 9:**
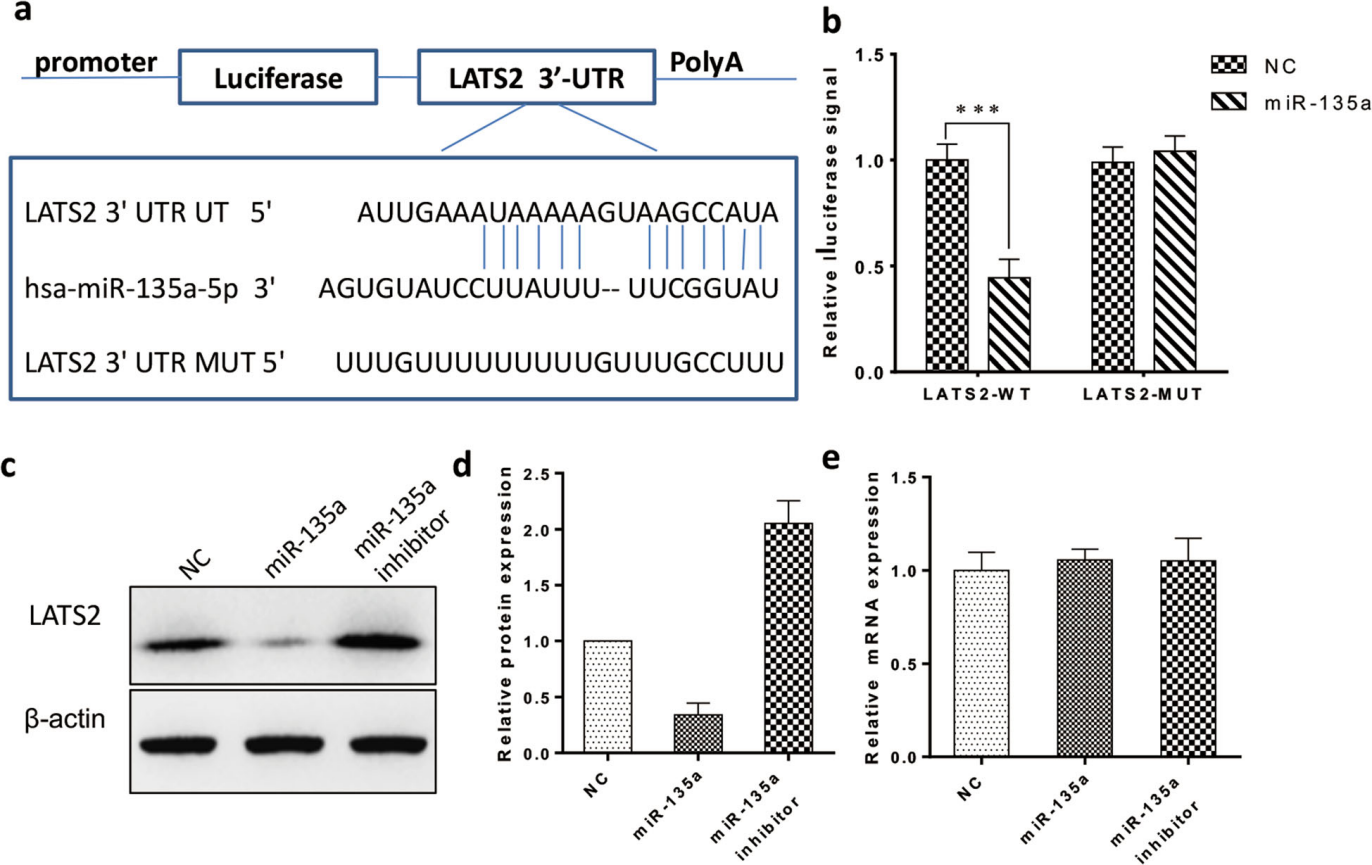
MiR-135a downregulated LATS2 by directly targeting the LATS2 3′ UTR. **a** Predicted miR-135a target sequences in the 3′UTR of LATS2 (LATS2-3′UTR). **b** Luciferase assays of fibroblasts transfected with LATS2-3′UTR. **c** Western blot of LATS2 expression after miR-135a overexpression or inhibition in fibroblasts. **d** Histogram summarizing the results in (**c**) (*n* = 3). **e** Relative LATS2 mRNA expression after miR-135a overexpression or inhibition in fibroblasts (*n* = 3)

We used the TargetScan and miRBase databases to predict the target genes of miR-135a by bioinformatics analysis and found that LATS2 is one of the candidate target genes. We constructed a luciferase reporter vector and demonstrated the interaction of miR-135a with the target gene LATS2 by the luciferase reporter assay (Fig. 9a, b). We also examined the expression of LATS2 protein and mRNA in fibroblasts overexpressing miR-135a and silenced for miR-135a. As shown, overexpression of miR-135a significantly reduced LATS2 protein expression, whereas inhibition of miR-135a increased LATS2 protein expression (Fig. 9d). Real-time quantitative PCR revealed that the mRNA level of LATS2 after overexpression or inhibition of miR-135a was not affected and was similar to that in the control group (Fig. 9e). These data indicated that miR-135a inhibits the translation of LATS2.

## Discussion

Human amnion mesenchymal stem cells (hAMSCs) are obtained by surgery from human amniotic tissue and are now often used as an alternative resource for stem cell therapy [13]. According to research reports, stem cells exert repair effects through paracrine signaling and affect cell proliferation and migration through the release of bioactive factors [14]. Exosomes are important paracrine factors secreted by numerous cell types, and current studies have found that they play a dominant role in tissue repair and regeneration [15]. Exosomes are also considered to be mediators of intercellular communication by transporting functional proteins or miRNAs to adjacent cells [16, 17]. We have found through experimental studies that artificially injected hAMSCs could increase the repair and healing of wounds in rats with skin lesions, and the same effect can be achieved by separately injecting exosomes extracted from hAMSCs. These findings are consistent with reports of human fibroblast-derived exosomes promoting wound healing in hereditary diabetic mice [18]. Similarly, studies have found that exosomes of human adipose-derived mesenchymal stem cells could promote skin wound healing [19]. However, the molecular mechanisms by which exosomes mediate wound repair and healing remain unclear. We found that miRNAs produced in hAMSC-Exos could promote cell proliferation and migration. Our animal experiments further confirmed that miRNAs produced in hAMSC-Exos significantly accelerated wound healing and repair in rats. Therefore, we speculated that the miRNAs produced in hAMSC-Exos could target corresponding mRNAs, thereby regulating the expression of proteins to promote cell proliferation and migration to accelerate wound healing.

miRNAs are a group of endogenous, highly conserved noncoding single-stranded RNAs of approximately 18–22 nt in length that are produced through DNA transcription but have no open reading frames and cannot be translated into proteins [20]. miRNAs were first discovered in 1933 by Lee in the embryonic development of nematodes, and the two miRNAs found were named lin-4 and let-7 [21]. Subsequent studies have found that miRNAs are widely found in animals and plants and participate in a variety of pathophysiological processes, such as growth, proliferation, development, wound healing, and tumorigenesis [22]. A mature miRNA binds to the complementary sequence on the 3′ UTR of its target mRNA and inhibits the translation of the mRNA or induces its degradation, thereby exerting a negative regulatory effect on gene expression at the posttranscriptional level. A single miRNA molecule can regulate the expression of multiple target genes, and a target gene can also be regulated by multiple miRNA molecules. The regulation of transcripts by miRNAs mainly occurs at the initial stage of translation, and the transcript of the target genes is destabilized by the action of capping and adenosine deaminase at the 5′ end [23]. miRNAs participate in a variety of pathological and physiological processes in the body and play an important role in the wound healing process, participating in various stages of the wound healing process [24–26]. Early studies of miR-135a have focused on tumor metastasis and inhibition [27, 28], and recent studies indicate that miR-135a can also promote epithelial cell migration by promoting EMT [29]. However, the role of miR-135a in wound healing remains unclear. The possible target genes of miR-135a in cell migration are still unclear. In the present study, we found a negative correlation between miR-135a and LATS2, that is, high levels of miR-135a were significantly associated with low levels of LATS2. miR-135a can significantly promote wound healing. In addition, overexpression of miR-135a accelerated cell migration in BJ cells in vitro, which was associated with miR-135a downregulating LATS2 expression.

In this study, we found that exosomal miRNAs produced by stem cells can play a role in wound healing in vitro, and we further studied the mechanism by which miRNAs promote wound healing. The study found that miR-135a accelerates cell migration by downregulating LATS2 levels to promote wound healing. This study provides a rationale for the therapeutic effect of exosomal miR-135a on wound healing. Compared to other wound treatments, exosomes are based on an endogenous mechanism that mimics intercellular communication, which provides a safer and more effective strategy. In conclusion, treatment based on hAMSC-Exos may be a candidate for the future promotion of wound healing, and the precise mechanism by which more miRNAs promote wound healing remains to be explored.

## Conclusions

In this study, we found that local injection of human amnion mesenchymal stem cells into wounds in rats could promote wound healing. Our results indicated that miRNAs produced by hAMSC-Exos could promote cell proliferation and migration. Additionally, miR-135a promoted wound healing, which may be mediated by the downregulation of LATS2 levels to accelerate cell migration. In addition, this study provides a rationale for the therapeutic effect on wound healing of miR-135a in exosomes derived from human amnion mesenchymal stem cells.

## Data Availability

All data generated or analyzed during this study are included in this published article.

## Abbreviations

hAMSC: Human amnion mesenchymal stem cell
hAMSC-Exo: Human amnion mesenchymal stem cell exosome
LATS2: Large tumor suppressor 2
α-SMA: Alpha-smooth muscle actin
hAMSC Exo miR-135 OE: miR-135-overexpressing human mesenchymal stem cell exosome
BJ cells: Human fibroblast BJ-1 cells
